# Incidence and Transmission of SARS-CoV-2 in US Child Care Centers After COVID-19 Vaccines

**DOI:** 10.1001/jamanetworkopen.2023.39355

**Published:** 2023-10-24

**Authors:** Timothy R. Shope, Khalil Chedid, Andrew N. Hashikawa, Emily T. Martin, Mary Ann Sieber, Gabrielle Des Ruisseau, John V. Williams, Sarah E. Wheeler, Monika Johnson, Myla Stiegler, Helen D’Agostino, G. K. Balasubramani, Kristin A. Yahner, Anna F. Wang-Erickson

**Affiliations:** 1 Division of General Academic Pediatrics, Department of Pediatrics, UPMC Children’s Hospital of Pittsburgh, University of Pittsburgh School of Medicine, Pittsburgh, Pennsylvania; 2 University of Michigan School of Public Health, Ann Arbor, Michigan; 3 Department of Emergency Medicine, Michigan Medicine, University of Michigan, Ann Arbor, Michigan; 4 Division of Pediatric Infectious Diseases, Department of Pediatrics, UPMC Children’s Hospital of Pittsburgh, University of Pittsburgh School of Medicine, Pittsburgh, Pennsylvania; 5 Institute for Infection, Inflammation, and Immunity in Children (i4kids), Pittsburgh, Pennsylvania; 6 Department of Pathology, University of Pittsburgh School of Medicine, UPMC, Pittsburgh, Pennsylvania; 7 University of Pittsburgh School of Public Health, Pittsburgh, Pennsylvania

## Abstract

**Importance:**

SARS-CoV-2 surveillance studies in US child care centers (CCCs) in the post–COVID-19 vaccine era are needed to provide information on incidence and transmission in this setting.

**Objective:**

To characterize SARS-CoV-2 incidence and transmission in children attending CCCs (students) and their child care providers (CCPs) and household contacts.

**Design, Setting, and Participants:**

This prospective surveillance cohort study was conducted from April 22, 2021, through March 31, 2022, and included 11 CCCs in 2 cities. A subset (surveillance group) of CCPs and students participated in active surveillance (weekly reverse transcription–polymerase chain reaction [RT-PCR] swabs, symptom diaries, and optional baseline and end-of-study SARS-CoV-2 serologic testing), as well as all household contacts of surveillance students. Child care center directors reported weekly deidentified self-reported COVID-19 cases from all CCPs and students (self-report group).

**Exposure:**

SARS-CoV-2 infection in CCC students.

**Main Outcomes and Measures:**

SARS-CoV-2 incidence, secondary attack rates, and transmission patterns were determined from diary entries, self-reports to CCC directors, and case logs. Incidence rate ratios were measured using Poisson regression clustering on centers with a random intercept and unstructured matrix.

**Results:**

From a total population of 1154 students and 402 CCPs who self-reported cases to center directors, 83 students (7.2%; mean [SD] age, 3.86 [1.64] years; 55 male [66%]), their 134 household contacts (118 adults [mean (SD) age, 38.39 (5.07) years; 62 female (53%)], 16 children [mean (SD) age, 4.73 (3.37) years; 8 female (50%)]), and 21 CCPs (5.2%; mean [SD] age, 38.5 [12.9] years; 18 female [86%]) participated in weekly active surveillance. There were 154 student cases (13%) and 87 CCP cases (22%), as defined by positive SARS-CoV-2 RT-PCR or home antigen results. Surveillance students had a higher incidence rate than self-report students (incidence rate ratio, 1.9; 95% CI, 1.1-3.3; *P* = .01). Students were more likely than CCPs to have asymptomatic infection (34% vs 8%, *P* < .001). The CCC secondary attack rate was 2.7% to 3.0%, with the upper range representing possible but not definite secondary cases. Whether the index case was a student or CCP, transmission within the CCC was not significantly different. Household cumulative incidence was 20.5%, with no significant difference in incidence rate ratio between adults and children. Household secondary attack rates were 50% for children and 67% for adults. Of 30 household cases, only 5 (17%) represented secondary infections caused by 3 students who acquired SARS-CoV-2 from their CCC. Pre- and poststudy seroprevalence rates were 3% and 22%, respectively, with 90% concordance with antigen or RT-PCR results.

**Conclusions and Relevance:**

In this study of SARS-CoV-2 incidence and transmission in CCCs and students’ households, transmission within CCCs and from children infected at CCCs into households was low. These findings suggest that current testing and exclusion recommendations for SARS-CoV-2 in CCCs should be aligned with those for other respiratory viruses with similar morbidity and greater transmission to households.

## Key Points

**Question:**

What are the SARS-CoV-2 cumulative incidence and transmission rates within child care centers (CCCs) and between CCCs and households?

**Findings:**

In this cohort study of 83 children in 11 CCCs and their household contacts (118 adults and 16 children) and child care providers (n = 21), SARS-CoV-2 cumulative incidence was 16.0%, and secondary attack rates at CCCs were 2.7% to 3.0% (with the upper range representing possible but not definite secondary cases) compared with 50% and 67% among household child and adult contacts, respectively. Of 30 household cases, only 5 (17%) were secondarily acquired from 3 students infected at the CCCs.

**Meaning:**

These findings suggest that children in CCCs play a small role in SARS-CoV-2 transmission into their households and that current testing and exclusion recommendations for CCCs should be aligned with those for other respiratory viruses with similar morbidity and greater transmission to households.

## Teaser

This cohort study characterizes SARS-CoV-2 incidence and transmission within child care centers and between centers and children’s households after SARS-CoV-2 vaccines became available.

## Introduction

Children younger than 5 years comprised a higher proportion of COVID-19 cases in the US in late 2021 and early 2022 than earlier in the pandemic.^1^ Children in group child care and schools can transmit many infectious diseases, particularly influenza,^2,3^ to household contacts and the community at large. COVID-19 mitigation efforts recommended by the Centers for Disease Control and Prevention (CDC) and local health departments have included closures of classrooms or entire child care centers (CCCs), mandatory SARS-CoV-2 testing in CCCs, and extended exclusion and quarantine periods to limit potential spread.^4^

A comprehensive understanding of the actual risk of SARS-CoV-2 transmission within CCCs and to households is vital for policy makers to implement appropriate, proportionate mitigation measures in the event of a surge or the emergence of new variants. However, there have been few reported SARS-CoV-2 surveillance studies of CCCs and none of US CCCs.^5,6,7^ We conducted a prospective, 12-month surveillance study to describe the incidence and transmission of SARS-CoV-2 among children (students) in CCCs, their household contacts, and center child care providers (CCPs).

## Methods

### Study Setting and Recruitment

This cohort study was conducted from April 22, 2021, through March 31, 2022, in 11 CCCs, including 5 in Pittsburgh, Pennsylvania, and 6 in Ann Arbor, Michigan. Four centers were university affiliated, and 7 were private. Throughout the study, COVID-19 vaccines were available for adults but not for children younger than 5 years. In November 2021, 5-year-old children became eligible to receive the COVID-19 vaccine. The study received approval from the institutional review boards at the University of Michigan Medical School (HUM00188466) and University of Pittsburgh (STUDY20070411) and followed the Strengthening the Reporting of Observational Studies in Epidemiology (STROBE) reporting guideline for cohort studies.^8^ With approval from CCC directors, the study team disseminated information to parents and CCPs, obtained their oral informed consent, and registered them using videoconferences.

### Participants

**Figure 1.**
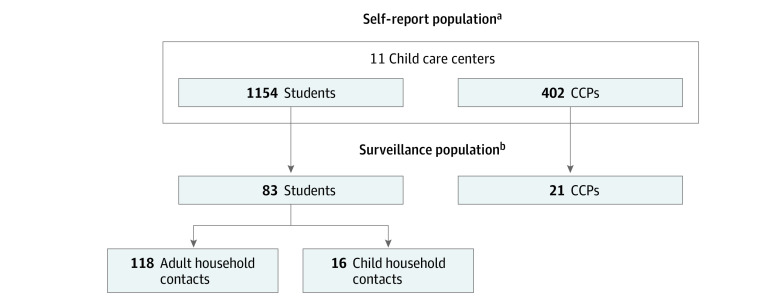
Composition and Data Collection of Self-Report and Surveillance Populations ^a^Pool of individuals who contributed person-time and case counts by self-report to the center directors, according to the centers’ policies. Center directors collated weekly self-reports of positive SARS-CoV-2 test results (antigen and reverse transcription–polymerase chain reaction) and presumptive positive diagnoses of students and child care providers (CCPs) in a child care center. Date of positive test result (if applicable), date of symptom onset (if applicable), and classrooms contacted were collected. Data were deidentified for students and CCPs not enrolled in the surveillance population. From this pool, a subset enrolled in the surveillance portion of the study. ^b^Pool of individuals in the active surveillance population recruited from the self-report population. Contributed weekly SARS-CoV-2 testing and symptom diaries. Optional SARS-CoV-2 serology testing of students, their household contacts, and CCPs was collected.

From the total pool of CCPs and students (self-report group) at the 11 CCCs, we recruited participants for weekly active surveillance (surveillance group) (Figure 1). Once a student was enrolled, all adult and child household contacts of the student were also included in the active surveillance population. Eligible students were younger than 6 years, attended the CCC 2 or more full days per week, and had 2 or more additional household members. Eligible CCPs worked at the CCC for a minimum of 2 days per week and had 15 or more minutes of close contact with children per day. Household adults and children were eligible if they spent more than 2 nights per week in the residence of the child attending the CCC. For consent and data collection feasibility, exclusion criteria included non–English-speaking individuals, parents younger than 18 years, or nonbiological parents. Rolling enrollment occurred from April 22, 2021, to March 31, 2022. When students aged out of the CCC, they either left the study (along with their household contacts) or entered the household group if they had siblings remaining in the CCC. Children in both student and child household groups were accounted for by incidence rates that incorporated the person-time they contributed to each group.

The self-report group was not enrolled. However, we obtained deidentified weekly director reports of COVID-19 cases at the CCCs. Generally, CCCs required individuals with new COVID-19 symptoms or following SARS-CoV-2 exposure to test and self-report, requiring negative test results prior to return to the center regardless of symptoms. We removed cases that were double counted by surveillance and director reports during data analysis.

### Data Collection

Child care center directors completed a registration survey, including contact information; number of classrooms, CCPs, and students; percentage of students who qualified for federally subsidized meals; and COVID-19 mitigation strategies (Table 1; eTable 2 in Supplement 1). Directors were surveyed monthly to identify changes in COVID-19 mitigation strategies and room attendance for surveillance participants. For all students and CCPs with COVID-19, directors provided symptom onset, positive test date, exclusion date, rooms visited by the infected individual while contagious, and the number of exposed CCP and student contacts in these rooms. After each case report, the study team monitored director reports for potential secondary cases and mapped transmission and directionality using prescribed case definitions and case logs. We determined the site (CCC, household, or other) of infection, distinguished between index and secondary cases, and noted the few indeterminate cases.

**Table 1.** Child Care Center Participation Rates and Qualification for Federally Subsidized Meals

Center No.	Child care providers		Students		Classrooms, total	Qualify for federally subsidized meals, %^c^
	Surveillance, No. (%)	Total^a^	Surveillance, No. (%)	Total^b^		
1	3 (5)	55	16 (12)	129	13	15
2	3 (7)	44	4 (4)	92	14	0
3	1 (3)	30	11 (11)	100	15	5
4	4 (10)	39	2 (2)	97	20	0
5	1 (7)	15	7 (15)	46	8	0
6	2 (7)	30	10 (14)	70	14	0
7	1 (3)	32	2 (3)	66	20	0
8	2 (5)	42	11 (8)	139	20	0
9	2 (11)	18	5 (7)	70	6	0
10	0 (0)	42	9 (6)	145	15	0
11	3 (5)	55	6 (3)	200	15	25
Total	22^d^ (6)	402	83 (7)	1154	160	6

^a^ Number of child care providers regularly employed. Number was assumed to remain constant during the study period.

^b^ Approximate number of children aged 0 to 5 years who attended 2 or more full days per week. Number was assumed to remain constant during the study period.

^c^ Percentage of children who receive federally subsidized meals through the US Department of Agriculture.

^d^ Includes 1 child care provider who moved to another participating center during the study and stayed in the study.

Surveillance participants provided demographic information (age, sex, and race and ethnicity), health history, and COVID-19 vaccine history. Participants also completed weekly diaries for reporting new COVID-19 symptoms, exposures or positive test results, and new COVID-19 vaccinations. Race and ethnicity were self-selected using a survey instrument and were included for study generalizability.

### Definitions

Exposure or contact was defined as being within 6 feet of an individual with COVID-19 for at least 15 minutes. The infectious period was the 2 days preceding and 10 days after the first symptom onset or positive test date, considered day 0. If exclusion occurred less than 10 days after day 0, the exposure period ended once children or CCPs were no longer in the CCC. A COVID-19–positive result was defined as any positive real-time, reverse transcription–polymerase chain reaction (RT-PCR) from surveillance swabs, diary entries, or antigen test from directors’ report.

An index case was defined as an individual with COVID-19 who transmitted the infection to an exposed person directly or indirectly. If a cluster occurred, the index case was the first person with day 0 criteria and separated by 1 or more days from any secondary cases. A secondary case was an individual with COVID-19 whose onset of symptoms or positive test result was 1 or more days after exposure to another individual with COVID-19 during their infectious period.

Incidence was defined as the number of new COVID-19 infections per total population. Secondary attack rate (SAR) was the number of new cases among contacts divided by the total number of contacts. Person-time was the number of days a person was at risk of contracting COVID-19 during the study period. A person was not considered at risk for 90 days after day 0 of infection.

### Nasal Swab Collection and Processing

Surveillance participants self-collected and submitted anterior nasal swabs weekly (PurFlock Ultra 6-inch sterile standard flock swab [Puritan Medical Supplies] or FLOQSwabs [Copan Diagnostics Inc]) using diagrammatic and written instructions. Swabs were placed in transport medium (Universal Viral Transport Medium [BD, Hardy Diagnostics] or Universal Transport Medium [Copan Diagnostics]), refrigerated by households, and transported via courier on ice within 48 hours to the laboratory for storage at −80 °C. Samples were aliquoted and stored at −80 °C. RNA was extracted using 200 μL of sample and eluted in 90 μL of elution buffer using the Automated ABI MagMax-96 Purification System (Thermo Fisher Scientific). Specimens were tested by validated CDC RT-PCR assays.^9^ Ann Arbor specimens were pooled in batches of 3, with deconvolution and individual testing for any positive pools.

### SARS-CoV-2 Serology

Participants provided optional blood specimens by venipuncture at our research clinics at the beginning and end of the study to determine their SARS-CoV-2 antibody status. Anti–SARS-CoV-2 nucleocapsid total antibodies were measured in Clinical Laboratory Improvement Amendments–certified University of Pittsburgh Medical Center clinical laboratories using the Elecsys Anti–SARS-CoV-2 double-antigen sandwich emergency use authorization assay (cobas e411; Roche Diagnostics) according to the manufacturer’s instructions.

### Statistical Analysis

Descriptive statistics were used for demographic information, case counts, and SARS-CoV-2 transmission rates within CCCs and households. Fisher exact and χ^2^ tests were used for comparing proportions. Poisson regression clustering on centers or households with a random intercept and unstructured matrix was used to compare incidence rates. Person-time included the time from the CCC registration to the end of the study. A 2-sided *P* < .05 was considered statistically significant. Statistical analyses were performed using SAS, version 9.4 (SAS Institute Inc) and Excel, version 18 (Microsoft Corporation). To address the effect of potential ascertainment bias on SARs, a post hoc sensitivity analysis was run assuming that incidence rates, frequencies of being symptomatic or asymptomatic, and proportion of secondary cases in self-report students were the same as in surveillance students.

## Results

### Surveillance Population

From a total population of 1154 students and 402 CCPs from 11 CCCs who self-reported cases to center directors, 83 students (7.2%; mean [SD] age, 3.86 [1.64] years; 28 female [34%] and 55 male [66%]), their 134 household contacts (118 adults [mean (SD) age, 38.39 (5.07) years; 62 female (53%), 55 (47%) male, and 1 preferring not to report their sex (1%)] and 16 children [mean (SD) age, 4.73 (3.37) years; 8 female (50%) and 8 male (50%)]), and 21 CCPs (5.2%; mean [SD] age, 38.5 [12.9] years; 18 female [86%] and 3 male [14%]) participated in weekly active surveillance (Table 1; Figure 1; eTable 1 in Supplement 1). Surveillance participants were primarily White (students: 68 White [82%], 5 Black [6%], and 10 other race and ethnicity [12%]; household adults: 95 White [81%], 6 Black [5%], and 17 other race and ethnicity [14%]; household children: 10 White [63%], 2 Black [13%], and 4 other race and ethnicity [25%]; CCPs: 20 White [95%], 1 Black [5%], and 1 other race and ethnicity [5%]), and only 3 CCCs (27%) had children qualifying for federally subsidized meals (eTable 1 in Supplement 1; Table 1). Surveillance CCPs and adult household contacts were highly immunized against both COVID-19 and influenza (20 [95%] and 108 [92%], respectively) (eTable 1 in Supplement 1). Twenty-one surveillance students [25%] were vaccinated against both COVID-19 (after November 2021) and influenza, and 59 [71%] were vaccinated against influenza only (eTable 1 in Supplement 1).

### Mitigation Measures

Generally, CCC directors followed CDC guidance^4^ to mitigate the spread of SARS-CoV-2 infection. Throughout the pandemic, directors required high levels of environmental cleaning and disinfecting, hand hygiene, mask wearing, and adherence to exclusion and quarantine periods for ill and exposed children and CCPs (eTable 2 in Supplement 1).

### Data Quality

Once enrolled in the surveillance subset, more than 93% of participants remained until completion, with diary and swab completion rates greater than 95% and 83%, respectively. Swab submission rates within the recommended 48-hour window were 90% and 88% in the Pittsburgh and Ann Arbor cohorts, respectively.

### Incidence

**Figure 2.**
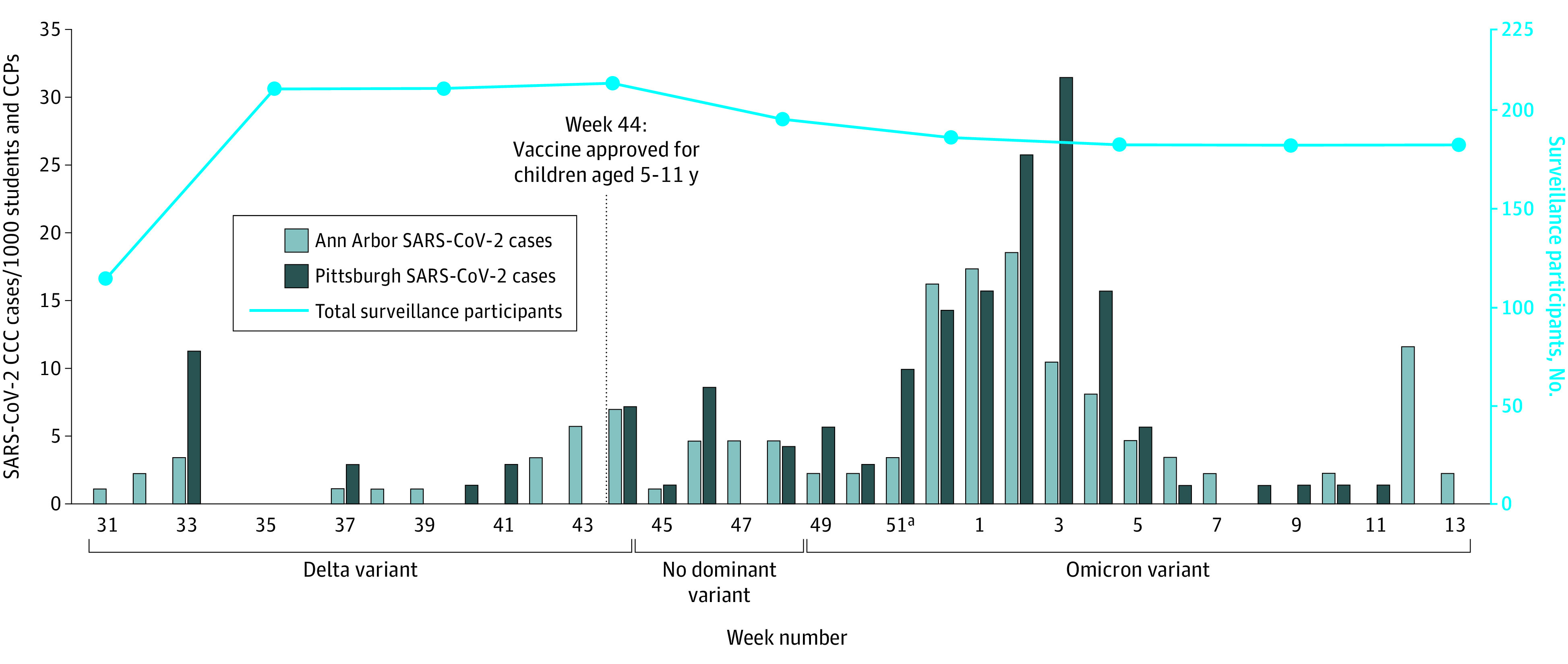
SARS-CoV-2 Incidence in Child Care Centers (CCCs) by Week During the Study Period Total cases among surveillance and self-report students and child care providers (CCPs) combined peaked after week 51 (winter break), when the Omicron variant was dominant in the US. Total enrollment of surveillance participants (CCPs, students, and household contacts) was fairly stable. The first reported case occurred in week 31, and preceding weeks are not shown.

There were 154 student cases (13%) and 87 CCP cases (22%), as defined by positive SARS-CoV-2 RT-PCR or home antigen results. The CCC incidence rates paralleled Ann Arbor and Pittsburgh and national surges of the dominant SARS-CoV-2 variants of concern over time (Figure 2). The highest incidence occurred just after the winter holiday break during the Omicron wave. Cumulative incidence varied significantly among centers, ranging from 6.8% (6 of 88 CCPs and students) to 26.5% (26 of 98 CCPs and students) (eTable 2 in Supplement 1). Among all surveillance participants, 50 of 238 (21.0%) had positive test results for SARS-CoV-2 during the study. The overall cumulative incidence was 16.0% (271 of 1690), including 2 self-report students with 2 cases each. The incidence rates per 10 000 person-days were 8.1 for all CCPs, 5.0 for all students (5.8 for CCPs and students combined), 10.3 for combined adult and child household contacts, and 6.1 for all individuals. Incidence rates were significantly higher for surveillance students than self-report students (incidence rate ratio [IRR], 1.9; 95% CI, 1.1-3.3; *P* = .01), but no significant difference was observed between surveillance and self-report CCPs (IRR, 1.1; 95% CI, 0.4-3.1; *P* = .82) (Table 2). Surveillance CCPs and students did not have significantly different incidence rates (IRR, 1.5; 95% CI, 0.5-4.7, *P* = .52), but self-report students had significantly lower incidence than self-report CCPs (IRR, 0.6; 95% CI, 0.4-0.7; *P* < .001) (eTable 3 in Supplement 1). Household cumulative incidence was 20.5%, and incidence rates were not significantly different between household adult and child contacts (IRR, 1.3; 95% CI, 0.4-3.5; *P* = .67) (Table 2).

**Table 2.** SARS-CoV-2 Incidence, Symptoms, and Transmission for All Participants (Self-Report) and Surveillance Subset

	Child care centers								Households, surveillance participants			
	Child care providers (n = 402)^a^				Students (n = 1154)^a^							
	Surveillance (n = 21)	Self-report (n = 402)^a^	IRR (95% CI)^b^	*P* value	Surveillance (n = 83)	Self-report (n = 1154)^a^	IRR (95% CI)^b^	*P* value	Adults (n = 118)	Children (n = 28)^c^	IRR (95% CI)^b^	*P* value
Person-days	4735	102 593			17 044	291 175			23 995	5042		
SARS-CoV-2–positive results (IR per 10 000 person-days)^d^	4 (8.4)	83 (8.1) [Reference]	1.1 (0.4-3.1)	.82	16 (9.4)	138 (4.7) [Reference]	1.9 (1.1-3.3)	.01	24 (10.0) [Reference]	6 (11.9)	1.3 (0.4-3.5)	.67
Asymptomatic infections (%)	0	7 (8)	NA	>.99	4 (25)	47 (34.1)^e^	NA	.58	4 (17)	2 (33)	NA	.57
Symptomatic infections (%)	4 (100)	76 (92)	NA	NA	12 (75)	91 (65.9)	NA	NA	20 (83)	4 (67)	NA	NA
Isolated cases min-max (%)^f^	2-3 (50-75)	61-64 (74-77)	NA	NA	8-9 (50-56)	82-91 (59.4-65.9)	NA	NA	6 (25)	1 (17)	NA	NA
Index cases min-max (%)^f^	0	11-13 (13-16)	NA	NA	1-2 (6-13)	15-20 (10.9-14.5)	NA	NA	2 (8)	2 (33)	NA	NA
Secondary cases min-max (%)^f^	1-2 (25-50)	8-9 (10-11)	NA	NA	6 (38)	32-36 (23.1-26.1)	NA	NA	16 (67)	3 (50)	NA	NA

Abbreviations: IR, incidence rate; IRR, incidence rate ratio; min-max, minimum to maximum; NA, not applicable.

^a^ Total population of all child care providers and students as reported by the center director, including those who later enrolled in the surveillance subgroup. All surveillance participants spent time in the self-report group.

^b^ Incidence rate ratio and *P* value calculated using Poisson regression clustering on centers with a random intercept and unstructured matrix.

^c^ Includes 12 students who started in the center surveillance subgroup, aged out, but stayed in the study as household children because they had a sibling who was still at a center.

^d^ Total cases of SARS-CoV-2 by reverse transcription–polymerase chain reaction or antigen test according to surveillance swab or reported by center director. Two students were each infected twice as reported by the center director.

^e^ Includes 1 positive case reported by a center director that did not have a symptomatic or asymptomatic designation.

^f^ Minimum definite cases and maximum cases, including possible cases that could not be determined with certainty, because of multiple exposures or matching onset dates, including (1) cases meeting the definition of an index case with the same date of illness onset of subsequent cases in the same classroom or household, (2) cases meeting the definition of an index case but for which secondary cases all had an additional exposure outside of the classroom or household, or (3) cases meeting the definition of a secondary case but with an exposure outside of the classroom or household. No household cases resulted in uncertain determination.

### Symptoms

Although COVID-19 symptoms were reported in weekly diary entries of 61 CCPs (9%), 532 students (21%), 805 adult household contacts (24%), and 86 child household contacts (17%), only 60 specimens (1%) were SARS-CoV-2 positive. No significant difference in asymptomatic cases was observed between surveillance and self-report CCPs (0 of 4 [0%] vs 7 of 83 [8%]; *P* > .99) or students (4 of 16 [25%] vs 47 of 138 [34%]; *P* = .58) or between household adult and child contacts (4 of 24 [17%] vs 2 of 6 [33%]; *P* = .57) (Table 2). Self-report student cases were more likely to be asymptomatic compared with self-report CCP cases (47 of 138 [34%] vs 7 of 83 [8%]; *P* < .001). However, surveillance CCPs and students were not significantly different due to a small sample size (0 of 4 [0%] vs 4 of 16 [25%]; *P* = .54) (eTable 3 in Supplement 1).

### Transmission

In the CCCs, most cases were isolated, with no differences in transmission whether the index case was a student or CCP (Table 2). When community spread was high, some surveillance participants were exposed to a person inside and outside the CCC on the same day, causing some uncertainty in secondary cases and SARs. Therefore, in Table 2 and Table 3, ranges were created, counting—or not counting—these double-exposed individuals as secondary cases.

**Table 3.** Secondary Attack Rates (SARs)^a^ of Child Care Providers, Students, and Household Surveillance Participants

	Child care centers				Households			
	Child care providers	Students	*P* value	Total	Adults	Children^b^	*P* value	Total
Total No. exposed^c^	409	1349	NA	1758	24	6	NA	30
Min-max cases (SAR, %)^d^	9-11 (2.2-2.7)	38-42 (2.8-3.1)	0	47-53 (2.7-3.0)	16 (67)	3 (50)	.64	19 (63)
No. exposed to adult cases	206	684	NA	890	7	3	NA	10
Min-max secondary cases due to adult contacts (SAR, %)^d^	4 (1.9)	14-16 (2.0-2.3)	1.0	18-20 (2.0-2.2)	3 (43)	1 (33)	1.0	4 (40)
No. exposed to child cases	203	665	NA	868	17	3	NA	20
Min-max secondary cases due to child contacts (SAR, %)^d^	5-7 (2.5-3.4)	24-26 (3.6-3.9)	.43	29-33 (3.3-3.8)	13 (77)	2 (67)	>.99	15 (75)

Abbreviations: Min-max, minimum to maximum; NA, not applicable.

^a^ Secondary attack rate is the number of new cases among contacts divided by the total number of contacts.

^b^ Includes household transmission of students and household child contacts.

^c^ Number of individuals exposed to child or adult isolated or index cases. Individuals were only counted once for an exposure to an index case; exposures to other secondary cases within the same classroom or household were not counted again.

^d^ Minimum definite cases and maximum cases, including possible cases that could not be determined with certainty because of multiple exposures or matching onset dates, including (1) cases meeting the definition of an index case but for which secondary cases all had an additional exposure outside of the classroom or household or (2) cases meeting the definition of a secondary case but with an exposure outside of the classroom or household.

The overall SAR in CCCs was between 2.7% and 3.0% (self-report student rate rose to 8.2% in the sensitivity analysis) but was much higher in households (50% for children and 67% for adults) (Table 3). Secondary attack rates did not significantly differ among adults and children, either within CCCs or households or whether the index case was an adult or a child. Only 3 of 51 (6%) students with asymptomatic infection (Table 2) transmitted the virus.

Surveillance students spent time in both CCCs and households, so we used case logs to determine the proportion of cases in the households caused by students who had acquired infection at the CCCs. Five of 30 (17%) household cases were caused by 3 symptomatic students infected at the CCCs. A fourth student was infected at the CCC but did not transmit to any household members. The remaining 12 of 16 (75%) student infections were acquired outside the CCC (n = 10) or were secondary cases of parents or siblings with infection in their household who did not go to the CCC (n = 2). When combining household cases (n = 30) and student cases (n = 16), only 9 of 46 (20%) that occurred in households were the result of student attendance at the CCC.

### Serology Results and RT-PCR or Antigen Positivity in Surveillance Population

During the study, seropositivity rose from 3% (4 of 138) to 22% (21 of 96) (eTable 4 in Supplement 1). Only 2 adult participants seroconverted without a positive RT-PCR or antigen test (90% concordance) (eTable 4 in Supplement 1).

## Discussion

This 12-month prospective cohort surveillance study revealed low SARS-CoV-2 transmission within CCCs. Specifically, when the Delta and early Omicron variants were predominant and before vaccines were available for children younger than 5 years, we found that children attending CCCs rarely transmitted the virus to their household contacts. To better describe transmission in CCCs, we augmented the relatively small surveillance population with weekly reports from CCC directors of the entire population. The surveillance subgroup findings validated the CCC directors’ reporting of SARS-CoV-2 cases for CCPs but indicated an undercounting among self-report students. Despite this limitation, we observed a cumulative incidence of 16% and low SARs (2.7%-3.0%, with self-report student SARs rising to 8.2% in sensitivity analysis) in the CCCs. These rates did not differ significantly between CCPs and students and did not differ based on whether the index case was a CCP or student. While asymptomatic infection was significantly higher in students than CCPs (34% vs 8%; *P* < .001), asymptomatic students rarely caused secondary infections. Despite high immunization rates, CCPs had higher incidence rates than students, possibly due to underdetection among self-report students.

Using only surveillance data, incidence rates in households were similar to those in CCCs, and there was no difference between children and adults. However, the SAR in households (50% for children and 67% for adults) was dramatically higher than in CCCs. Combining student cases (n = 16) and household cases (n = 30), because students spent time in both settings, only 9 of 46 (20%) cases in households were the result of student attendance at the CCC (4 students acquired infection at their CCCs, 3 of whom caused 5 of 30 household cases). Although students who acquired infection at CCCs were uncommonly the cause of household cases, they could still acquire infection from other sources; given the very high household SAR when a child was the index (75%) (Table 3), vaccination of young children could be beneficial.

Our study is, to our knowledge, the first published report to examine SARS-CoV-2 epidemiology in CCCs in the postvaccine era, reflecting the current US status in which less than 11% of children younger than 5 years have received at least 1 COVID-19 vaccination compared with more than 82% of adults.^10^ Previous studies of SARS-CoV-2 in CCCs were performed before vaccines, and most occurred in the context of outbreak or contact tracing investigations^11,12,13,14,15,16,17,18,19,20,21^ or during periods of low community transmission.^5,6,7^ Surveillance studies, with regular testing regardless of symptoms, may enable more accurate detection of asymptomatic infection and transmission. In contrast, the surveillance subset of our study sample yielded higher incidence rates only in students, not CCPs. Because there was no significant difference between surveillance and self-report student groups’ symptomatic and asymptomatic infection rates (Table 2), there may have been underdetection of asymptomatic as well as symptomatic children in the self-report group. Surveillance students had a high frequency of COVID-19–like symptoms (>20% diary reports) with less than 5% symptomatic infections caused by SARS-CoV-2. This high student symptom burden, which has since worsened with the resurgence of many viral respiratory pathogens, may mean that parents and CCC directors may not recognize which symptomatic children should be tested for SARS-CoV-2.

By February 2022, US SARS-CoV-2 seroprevalence was 68%^22^ in children aged 1 to 4 years, which is significantly higher than our end-of-study seroprevalence (22%). Previously published prevaccine studies in CCCs reported a range of SARs, from 0.5% to 9.6%^6,11,12,14,15,20,21^ to more than 20%.^13,16,17,18,19^ Secondary attack rates varied considerably in these prevaccine studies, possibly due to variable mitigation measures. Our study, with its longer duration (12 months) and weekly sampling, shows a striking difference in SARs between CCCs (approximately 3%) and households (63%), similar to 1 other report.^21^ A meta-analysis showed that household SARs increased to 42.7% during the Omicron wave in association with increasingly contagious variants and decreasing vaccine efficacy.^23^ The higher SARs in households likely reflect extended periods of exposure, closer contact, and minimal mitigation.

The lower CCC SARs observed in our study may be attributed to underdetection of self-report student cases as well as a combination of mitigation measures, including high adult immunization rates, cohorting, mask wearing, improved ventilation, enhanced cleaning and sanitizing, exclusion of ill children, testing, quarantining and isolation, and room closures after exposures. Because of the low number of cases, we could not model the association of overall mitigation measures, which largely followed CDC guidance^4^ and mask wearing specifically, with SARS-CoV-2 incidence.

During the early pandemic, there was a focus on minimizing potential transmission from children at CCCs to nonvaccinated and more vulnerable adult contacts. However, some of the mitigation efforts, such as prolonged exclusions, room closures, and CCC closures, were reported to have a detrimental effect on the financial viability of US child care programs^24^ and may have hindered parents’ ability to reenter or remain in the workforce.^25^

With widespread SARS-CoV-2 immunity from vaccines and prior infections and the expiration of the public health emergency, it is appropriate to reassess recommended exclusion criteria, which still include testing anyone with respiratory symptoms and staying home if positive for at least 5 days.^26^ Children with respiratory syncytial virus infection and influenza, both known to spread quickly within CCCs and to households^27,28,29,30^ and with similar morbidity to SARS-CoV-2,^2,3,31,32,33,34^ may return to CCCs once they are behaviorally able to participate without requiring excess care and after fever has resolved for 24 hours without antipyretics,^35,36^ which is often less than 5 days. Our findings support relaxing SARS-CoV-2 testing and exclusion recommendations for mildly symptomatic or exposed children.

### Strengths and Limitations

The strengths of this study include the prospective surveillance design with weekly testing at multiple sites, case logs to track transmission, low participant attrition, and high-quality data. The 12-month duration covered SARS-CoV-2 Delta and Omicron variants. Furthermore, including household contacts provided valuable insights into the directionality of transmission, which has important policy implications.

The study also had some limitations. The CCCs served relatively high-income families. The surveillance sample represented a small proportion of the overall CCC population, and because the self-report population comprised nonactive participants, we had no demographic information and may have underdetected student cases. The study’s generalizability is limited by the high vaccine rate in the surveillance population, unpredictable characteristics of future circulating SARS-CoV-2 variants, participants’ relative lack of prior SARS-CoV-2 immunity, and substantial mitigation measures in place during the study.

## Conclusions

This cohort study revealed that SARS-CoV-2 transmission within CCCs was low between May 2021 and March 2022. Unlike other viral causes of respiratory infections, children attending CCCs did not significantly contribute to SARS-CoV-2 transmission within the CCCs or to their household members. These findings suggest that current testing and exclusion recommendations for SARS-CoV-2 in CCCs should be aligned with those for other respiratory viruses with similar morbidity and greater transmission to households.

## Notes

Supplement 1. **eTable 1.** Surveillance Participant Demographics **eTable 2.** Child Care Center COVID-19 Mitigation Measures **eTable 3.** Incidence Rate Ratios at Child Care Centers for All Participants (Self-Report) and the Surveillance Subset **eTable 4.** SARS-CoV-2 Seropositivity and RT-PCR/Antigen Test Positivity

Supplement 2.
Data Sharing Statement
