# Supplementary material for: Incidence and Transmission of SARS-CoV-2 in US Child Care Centers After COVID-19 Vaccines
Source: JAMA Netw Open. 2023 Oct 24;6(10):e2339355. doi: 10.1001/jamanetworkopen.2023.39355 (PMC10599125; doi:10.1001/jamanetworkopen.2023.39355)
Supplement: Supplement 1. — eTable 1. Surveillance Participant Demographics eTable 2. Child Care Center COVID-19 Mitigation Measures eTable 3. Incidence Rate Ratios at Child Care Centers for All Participants (Self-Report) and the Surveillance Subset eTable 4. SARS-CoV-2 Seropositivity and RT-PCR/Antigen Test Positivity [file jamanetwopen-e2339355-s001.pdf]

Supplemental Online Content

Shope TR, Chedid K, Hashikawa AN, et al. Incidence and transmission of SARS-CoV-2 in US child care centers after COVID-19 vaccines. *JAMA Netw Open*. 2023;6(10):e2339355. doi:10.1001/jamanetworkopen.2023.39355

eTable 1. Surveillance Participant Demographics

eTable 2. Child Care Center COVID-19 Mitigation Measures

eTable 3. Incidence Rate Ratios at Child Care Centers for All Participants (Self-Report) and the Surveillance Subset

eTable 4. SARS-CoV-2 Seropositivity and RT-PCR/Antigen Test Positivity

This supplemental material has been provided by the authors to give readers additional information about their work.

© 2023 Shope TR et al. *JAMA Network Open.*

eTable 1. Surveillance Participant Demographics

	Surveillance students (n=83)	Surveillance CCPs (n=21)	Household Children (n=16)[a]	Household Adults (n=118)
Age in years[b]	3.86 (1.64)	38.5 (12.9)	4.73 (3.37)	38.39 (5.07)
Sex				
Female	28 (34)	18 (86)	8 (50)	62 (52.5)
Male	55 (66)	3 (14)	8 (50)	55 (46.6)
Prefer not to Answer	0 (0)	0 (0)	0 (0)	1 (0.8)
Race[c]				
White	68 (82)	20 (95)	10 (63)	95 (80.5)
Black	5 (6)	1 (5)	2 (13)	6 (5.1)
Other	10 (12)	1 (5)	4 (25)	17 (14.4)
Ethnicity				
Hispanic	2 (2)	0 (0)	0 (0)	2 (1.7)
Not Hispanic	80 (96)	20 (95)	16 (100)	116 (98.3)
Unknown/Prefer not to Answer	1 (1)	1 (5)	0 (0)	0 (0)
Vaccination Status[d]				
Influenza only	59 (71)	0 (0)	8 (50)	0 (0)
COVID only	0 (0)	1 (5)	0 (0)	10 (8.5)
Both	21 (25)	20 (95)	6 (38)	108 (91.5)
Neither	3 (4)	0 (0)	2 (12)	0 (0)
Clinical Site				
Pittsburgh	41 (49)	10 (48)	9 (56)	60 (50.8)
Ann Arbor	42 (51)	11 (52)	7 (44)	58 (49.2)

Abbreviation: CCP, child care provider.
Mean (standard deviation) for continuous variables and No. (%) for categorical variables
[a]Does not include 12 students who later became child household contacts
[b]Age was calculated at the end of the study due to vaccination status being updated throughout the study.
[c]Participants could select more than one option
[d]At least one dose during the study period

© 2023 Shope TR et al. *JAMA Network Open.*

eTable 2. Child Care Center COVID-19 Mitigation Measures

Center		Temperature check	Physical distancing	Masking[a]		Enhanced cleaning	Hand hygiene	Exclusion of ill children	CCP COVID vaccine rate, %	COVID cumulative incidence (%)[b]
				Students	CCPs					
1	Start (04/22/21)	3	1	2	3	3	3	3	88	32/184 (17.4)
	Sep	0	1	3	3	3	3	3	93	
	Dec	0	2	2	3	3	3	3	98	
	Mar	0	1	3	3	3	3	3	--	
2	Start (05/21/21)	3	1	3	3	3	3	3	--	18/136 (13.2)
	Sep	3	1	2	2	3	3	3	92	
	Dec	3	1	2	2	3	2	3	93	
	Mar	2	1	1	1	3	2	3	95	
3	Start (05/21/21)	3	0	3	3	3	3	3	100	20/130 (15.4)
	Sep	1	1	1	3	3	3	3	100	
	Dec	1	1	1	3	3	3	1	100	
	Mar	1	1	1	1	3	3	1	100	
4	Start (05/28/21)	3	1	2	2	3	3	3	100	24/136 (17.6)
	Sep	1	1	2	2	3	3	3	100	
	Dec	1	1	2	2	3	3	3	100	
	Mar	1	1	2	2	3	3	3	100	
5	Start (06/01/21)	3	2	3	3	2	3	3	72	13/61 (21.3)
	Sep	3	2	3	3	3	3	3	72	
	Dec	3	2	3	3	3	3	3	83	
	Mar	3	2	3	3	3	3	3	84	
6	Start (06/15/21)	3	1	3	3	2	2	3	100	16/100 (16.0)
	Sep	3	0	2	3	2	2	3	100	
	Dec	3	2	2	3	2	2	3	100	
	Mar	3	1	2	3	2	2	3	100	
7	Start (06/23/21)	3	3	3	3	3	3	3	99	26/98 (26.5)
	Sep	3	2	3	3	3	3	3	100	
	Dec	3	3	3	3	3	3	3	100	
	Mar	3	1	3	3	3	3	3	100	
8	Start (07/08/21)	3	1	2	2	3	3	3	100	40/181 (22.1)
	Sep	3	1	3	3	3	3	3	100	
	Dec	3	1	3	3	3	3	3	100	
	Mar	3	2	3	3	3	3	3	100	

© 2023 Shope TR et al. *JAMA Network Open.*

9	Start (07/08/21)	3	3	3	3	3	3	3	100	6/88 (6.8)
	Sep	3	3	3	3	3	3	3	100	
	Dec	3	3	3	3	3	3	3	100	
	Mar	3	3	3	3	3	3	3	100	
10	Start (07/12/21)	3	1	2	3	2	3	3	100	19/187 (10.2)
	Sep	3	1	2	3	2	3	3	100	
	Dec	3	1	2	3	2	3	3	100	
	Mar	3	2	1	1	1	3	3	100	
11	Start (9/16/21)	3	2	2	3	3	3	3	100	27/255 (10.6)
	Sep	3	2	2	3	3	3	3	100	
	Dec	3	1	3	3	3	3	3	100	
	Mar	3	3	2	3	3	3	3	100	

Abbreviations: CCP, child care provider; COVID-19, coronavirus infectious disease 2019
Key: 0=never; 1=sometimes; 2=most of the time; 3=always; Start, child care center study start date (month/day/year); Sep, September; Dec, December; Mar, March; -- Missing data
[a]Masks were not worn during meals, snacks, or naps. Children <2 years did not mask. Some rooms had mixed masking due to transitional ages or to consolidate classrooms at the beginning or end of the day
[b]COVID-19 cumulative incidence in each center (CCPs and students combined)

© 2023 Shope TR et al. *JAMA Network Open.*

eTable 3. Incidence Rate Ratios at Child Care Centers for All Participants (Self-Report) and the Surveillance Subset

	**Surveillance CCPs (n=21)**	**Surveillance Students (n= 83)**	**IRR (95% CI)[a]**	***P***	**Self-Report CCPs (n=402)[b]**	**Self-Report Students (n=1154)[b]**	**IRR (95% CI)[a]**	***P***
SARS-CoV-2 Positives (IR/10,000 person-days)	4 (8.4) ref	16 (9.4)	1.5 (0.5, 4.7)	0.5[a]	83 (8.1) ref	138 (4.7)	0.6 (0.4, 0.7)	0.0001[a]
Asymptomatic (%)	0 (0)	4 (25)	NA	0.5	7 (8)	47 (34.1)	NA	<0.0001
Symptomatic (%)	4 (100)	12 (75)	NA	NA	76 (92)	91 (65.9)	NA	NA

Abbreviations: CCP, child care provider; IR, incidence rate; IRR, incidence rate ratio; CI, confidence interval; *P*, *P*-value; NA, not applicable; ref, reference group.

[a]Incidence rate ratio and *P* value calculated using Poisson regression clustering on centers with a random intercept and unstructured matrix.

[b]Total population of all CCPs and students as reported by the center director at registration, including those who later enrolled into the surveillance subgroup. All surveillance participants spent time in the self-report group.

© 2023 Shope TR et al. *JAMA Network Open.*

eTable 4. SARS-CoV-2 Seropositivity and RT-PCR/Antigen Test Positivity

	Centers		Households		
	CCPs n=21	Students n=83[a]	Adults n=118	Children n=28[a]	Total n=238
Baseline Positive (%)[b]	2/19 (11)	2/23 (9)	0/92 (0)	0/4 (0)	4/138 (2.9)
End-of-study Positive (%)[c]	4/15 (27)	1/15 (7)	15/63 (24)	1/3 (33)	21/96 (22)
PCR/Antigen Positive[d]	2	1	15	1	19
Concordance (%)	2/4 (50)	1/1 (100)	15/15 (100)	1/1 (100)	19/21 (90)

Abbreviations: SARS-CoV-2, severe acute respiratory syndrome coronavirus 2; RT-PCR, real-time reverse transcription polymerase-chain reaction; CCPs, child care providers

[a]Includes 12 students who aged out of the child care center, but stayed in the study as a household child because they had a sibling who was still at a child care center. These students contributed to the student baseline positive and the household child end-of-study positive categories.

[b]Participants who had blood drawn at the beginning of the study

[c]Participants who had blood drawn at the end of the study

[d]Only among participants who had an end-of-study blood draw

© 2023 Shope TR et al. *JAMA Network Open.*
