# Supplementary material for: Incidence and Transmission of SARS-CoV-2 in US Child Care Centers After COVID-19 Vaccines
Source: JAMA Netw Open. 2023 Oct 24;6(10):e2339355. doi: 10.1001/jamanetworkopen.2023.39355 (PMC10599125; doi:10.1001/jamanetworkopen.2023.39355)
Supplement: Supplement 2. — Data Sharing Statement [file jamanetwopen-e2339355-s002.pdf]

Data Sharing Statement

Shope. Incidence and Transmission of SARS-CoV-2 in US Child Care Centers After COVID-19 Vaccines. *JAMA Netw Open*. Published October 24, 2023.
doi:10.1001/jamanetworkopen.2023.39355

Data
Data available: No

Additional Information
Explanation for why data not available: Ongoing different study using the data
